# Characteristics and Survival of Intensive Care Unit Patients with Coronavirus Disease in Osaka, Japan: A Retrospective Observational Study

**DOI:** 10.3390/jcm10112477

**Published:** 2021-06-03

**Authors:** Ling Zha, Tomotaka Sobue, Taro Takeuchi, Kenta Tanaka, Yusuke Katayama, Sho Komukai, Atsushi Hirayama, Takeshi Shimazu, Tetsuhisa Kitamura, the COVID-19 Epidemiology Research Group of Osaka University

**Affiliations:** 1 Environmental Medicine and Population Sciences, Department of Social Medicine, Graduate School of Medicine, Osaka University, 2-2 Yamadaoka, Suita 565-0871, Japan; tsobue@envi.med.osaka-u.ac.jp (T.S.); tarogauss106072@gmail.com (T.T.); tanaken.0414@gmail.com (K.T.); lucky_unatan@yahoo.co.jp (T.K.); 2 Traumatology and Acute Critical Medicine, Department of Acute Critical Medicine, Graduate School of Medicine, Osaka University, 2-2 Yamadaoka, Suita 565-0871, Japan; orion13@hp-emerg.med.osaka-u.ac.jp (Y.K.); shimazu@hp-emerg.med.osaka-u.ac.jp (T.S.); 3 Biomedical Statistics, Department of Integrated Medicine, Graduate School of Medicine, Osaka University, 2-2 Yamadaoka, Suita 565-0871, Japan; skomukai@biostat.med.osaka-u.ac.jp; 4 Public Health, Department of Social Medicine, Graduate School of Medicine, Osaka University, 2-2 Yamadaoka, Suita 565-0871, Japan; atsushihirayamamd@gmail.com

**Keywords:** intensive care unit (ICU), age, mortality, COVID-19, survival analysis, Japan

## Abstract

The epidemiological and clinical characteristics, treatments, and outcomes of patients with coronavirus disease 2019 (COVID-19) who are admitted to the intensive care unit (ICU) have not been adequately evaluated in Japan. We analyzed the registry data of 205 patients with COVID-19 admitted to the ICU between February and November 2020, in Osaka Prefecture, Japan. A Cox proportional hazards model was used to assess the association between epidemiological factors and mortality among ICU patients. Of the 205 ICU patients, 161 (78.5%) were men and 149 (72.7%) were older than 60 years. A total of 117 patients (57.1%) had comorbidities. The most common symptoms at diagnosis were mild (*n* = 131, 63.9%). A total of 187 patients (91.2%) received mechanical ventilation, and 32 patients (15.6%) required extracorporeal membrane oxygenation. Patients were followed up for a median of 25 days after ICU admission. A total of 147 patients (71.7%) were alive at discharge, and 58 patients (28.3%) died. The hazard ratio for mortality among patients aged >80 years was 6.02 (95% confidence interval: 2.10−17.25) in the multivariable model, which was higher than that among those aged ≤59 years. These results are useful for recognizing the clinical course of this infection in ICU patients.

## 1. Introduction

The current coronavirus disease (COVID-19) pandemic is a public health emergency of international concern and poses immense challenges to healthcare systems, including increased rates of hospital admissions and demand for intensive care unit (ICU) beds, advanced respiratory support, and trained healthcare professionals [1]. The ICU receives critically ill patients and supports them through their critical phase of life. As of 23 March 2021, 124,364,349 severe acute respiratory syndrome coronavirus 2 (SARS-CoV-2) infections and 2,737,382 COVID-19-related deaths have been reported worldwide [2]. Among the active cases, 0.4% (90,596 of 21,275,893) were in severe or critical conditions [2]. According to previous overseas studies, approximately 10–15% of COVID-19 patients required hospitalization, and 20–30% of hospitalized patients developed severe or life-threatening symptoms [3]. The reported mortality rate of COVID-19 patients is 20–40% among hospitalized patients and 30–88% among critically ill and ICU patients, and this varies widely across countries and regions [3,4,5].

Osaka Prefecture, the largest region of western Japan, was the second epicenter of the COVID-19 outbreak in Japan. The first COVID-19 case in Japan was confirmed on 16 January 2020, and by 22 March 2021, 9,324,869 confirmed cases were reported nationwide [6]. Of these, 49,420 cases were confirmed in Osaka Prefecture [6]. Though several studies have reported the characteristics and factors related to mortality among ICU patients with COVID-19 overseas, the current situation of ICU patients in Japan based on regional registration is not clearly understood [7]. Therefore, we conducted this study to elucidate the characteristics of patients with COVID-19 who required ICU admission and the actual situation in the ICU as well as the factors related to the outcome of these patients using the COVID-19 patient registry managed by Osaka Prefecture, Japan.

## 2. Materials and Methods

### 2.1. Population, Design, and Setting

This retrospective study was conducted in Osaka Prefecture, Japan, from January 2020 to November 2020. In Osaka Prefecture, based on the Infectious Diseases Control Law, an active epidemiological investigation on COVID-19 was conducted to collect epidemiological information on COVID-19 patients using a data collection system with a uniform format managed by Osaka Prefecture [8]. Data collection was conducted daily via telephone or secured email. All cases were registered using this system until October 2020. In November 2020, this system and the government-recommended system were used together, and this system fully transitioned to the government-recommended system (Health Center Real-time Information-sharing System on COVID-19: HER-SYS) [9]. Therefore, not all cases were registered in November 2020.

Osaka Prefecture is located in the central area of western Japan and covers an area of 1905 km^2^. The estimated population was 8,819,226 on 1 April 2020 [10]. Local public health centers collected data telephonically or via worksheets maintained for all laboratory-confirmed COVID-19 patients. All COVID-19 cases diagnosed at hospitals or testing centers were reported to the local health center. The directors of local public health centers decided on the patients’ treatment course (hospitalization, recuperation at accommodation facilities, or recuperation at home) based on their vital signs, symptoms, age, and comorbidities. The collected data were reported to the Osaka Prefectural Government and the Ministry of Health, Labor, and Welfare [11]. According to the Ministry of Health, Labor, and Welfare of Japan, the release criteria from isolation for symptomatic patients with COVID-19 were as follows: 10 days after the onset of symptoms; at least 3 days without any symptoms, including fever and/or respiratory-related symptoms; or two consecutive negative laboratory results, such as the polymerase chain reaction test conducted at least 24 h apart [9]. In addition, the release criteria for the follow-up of asymptomatic patients were 10 days after a positive COVID-19 test result or two consecutive negative results at least 24 h apart 6 days after the positive test results.

Of the COVID-19 cases enrolled by the data collection system from January 2020 to November 2020, we received cases only with complete follow-up history from Osaka Prefecture.

The study was conducted in accordance with the Declaration of Helsinki, and the protocol was approved by the Research Ethics Committee of Osaka University (Approval No. 20397).

### 2.2. Measurements

Information on COVID-19 cases included age, gender, comorbidities, residence, cluster, close contacts, symptoms at diagnosis, hospitalization, death, date of symptom onset, date of hospitalization, date of death (for patients who died during the observation period), and in-hospital treatments (oxygen therapy; invasive/non-invasive mechanical ventilation (MV); and extracorporeal membrane oxygenation (ECMO); ICU admission; and dialysis). The age groups were clustered for every 10 years in the available data. ICU patients were defined as those having information of either ICU admission or ICU discharge date.

In accordance with the definitions outlined by Osaka Prefecture, we defined the surge according to the onset date as follows: the first surge (until 13 June 2020), the second surge (from 14 June 2020 to 9 October 2020), and the third surge (after 10 October 2020) [12]. High-risk comorbidities, such as diabetes; heart failure; respiratory diseases, including chronic obstructive pulmonary disease (COPD); chronic kidney diseases requiring dialysis; and the use of immunosuppressants and anticancer drugs, were summarized in this database [12]. A cluster was a group of five or more COVID-19 positive cases with an epidemiological link to the primary identified COVID-19 cases identified from various facilities, such as nursing homes, medical institutions, and restaurants [13]. Close contacts were identified by local public health centers as those who lived with the confirmed cases or had prolonged contact with the patients; those who examined, nursed, or cared for the confirmed cases without personal protection equipment; those who were likely to have had direct contact with contaminated materials; or those who had contact with confirmed cases for >15 min at a short distance (approximately 1 m) without personal protection equipment [13]. Symptoms at diagnosis were defined as follows: asymptomatic, mild (only cough without breathlessness or respiratory symptoms), moderate (breathlessness, pneumonia, or necessity of oxygen therapy), and severe (necessity of stay in the ICU or the use of MV) [11]. Cluster in this study was categorized as follows: no, medical institution, and others. The onset date was defined as the date when symptoms were estimated to have appeared [14]. If the onset date was missing, we substituted the onset date for the date of medical treatment, the date of hospital admission, or the date when a change in symptoms was noted, whichever occurred first. Hospitalized patients had any of the following items: date of hospitalization and/or discharge or reason of discharge (alive or death).

### 2.3. Outcomes and Follow-Up Period

The main outcome of this study was mortality during the observational period. The follow-up period started at the onset date and was censored at the end of follow-up or the date of death, whichever occurred first.

### 2.4. Statistical Analysis

Categorical variables are presented as frequencies and percentages. Continuous variables are presented as medians and interquartile ranges (IQRs). We assessed the age group trends at baseline and clinical characteristics using linear trend tests. The distribution of the number of COVID-19 patients by age groups was described by weeks.

The treatment period was calculated using the initiation and termination dates for each treatment. Considering that the onset date was equal to the end of follow-up or the initiation date of each treatment was equal to the termination date (i.e., 0 days of follow-up), we added 1 day to the observation period. In this study, we defined the end of follow-up as the death date or the last-known-alive date such as the date of hospital discharge or the date when the release criteria were met.

The epidemiological and clinical characteristics were compared between survivors and deaths by using the Pearson’ s chi-squared test or Fisher’ s exact test for categorical variables and Wilcoxon rank-sum test for continuous variables as appropriate.

To assess the association between factors and mortality among ICU patients with COVID-19, univariable and multivariable Cox regression analyses were used to estimate the hazard ratios (HRs) and 95% confidence intervals (CIs). The multivariable model was adjusted for gender (male or female), age (0−59, 60−69, 70−79, and ≥80 years), comorbidities (yes or no), area of residence (Osaka City, other cities), symptoms at diagnosis (mild, moderate to severe, or unknown), surge (first, second, or third), cluster (yes or no), and close contact (yes or no). All tests were two-tailed, and statistical significance was set at *p* < 0.05. All statistical analyses were performed using STATA version 16.0MP (STAT Corp., College Station, TX, USA).

## 3. Results

### 3.1. Eligible Patients

**Figure 1:**
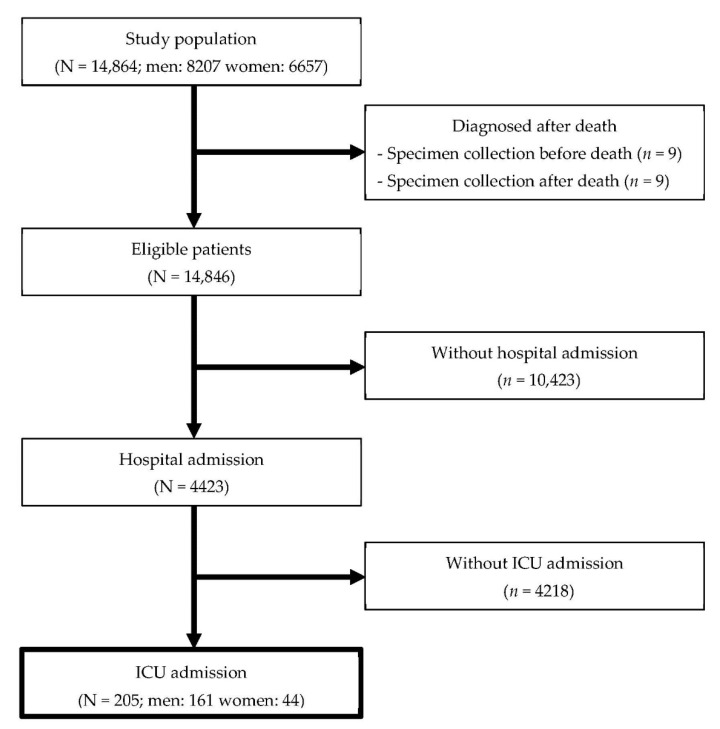
Flow diagram of patients admitted to the intensive care unit.

Of the 14,846 eligible patients with COVID-19 who were registered in Osaka Prefecture during the study period, 4423 patients (29.8%) were hospitalized and 205 patients (4.6% of hospitalized patients) were admitted to the ICU in 26 hospitals. A total of 205 COVID-19 patients (men, 161; women, 44) were included in the present study (Figure 1), registered between 17 February and 5 November 2020. Of the 205 patients, one had a missing ICU admission date and 40 had a missing ICU discharge date. Patients were followed up for a median of 25 days after ICU admission. In total, 147 patients (71.7%) were alive at discharge, and 58 patients (28.3%) died in the ICU.

### 3.2. The Description of Baseline Features

The baseline epidemiological characteristics of patients admitted to the ICU are presented in Table 1. Critically ill patients included 161 men (78.5%), and 149 patients (72.7%) were older than 60 years. Most ICU patients lived in Osaka City (*n* = 128, 62.4%). More than half of the patients (*n* = 117, 57.1%) had comorbidities. The most common symptoms at diagnosis were mild (*n* = 131, 63.9%), followed by severe symptoms (*n* = 69, 33.7%), and there were no asymptomatic cases among ICU patients. There were 116 cases (56.6%) during the first surge, 67 cases (32.7%) during the second surge, and 22 cases (10.7%) during the ongoing third surge.

### 3.3. The Description of Clinical Features

Table 2 shows the clinical characteristics of ICU patients with COVID-19 in Osaka Prefecture. Of the 205 patients, 147 (71.7%) were discharged alive and 58 (28.3%) died during ICU admission. The median duration from the onset of symptoms to ICU admission among patients who died was 8 days (IQR: 5−11 days), which was significantly shorter than that among patients who survived (10 days (IQR: 8−12 days), *p* = 0.023). The median length of ICU stay was 13 days (IQR: 9−21 days) for patients who survived and 18 days (IQR: 9−28 days) for patients who died (*p* = 0.177). A total of 187 patients (91.2%) required MV, including non-invasive positive pressure ventilation, with a median duration of 13 days (IQR: 8−23 days). The median duration of MV for patients who died (17 days (IQR: 10−26.5 days)) was significantly longer than that for patients who survived (13 days (IQR: 8−22 days), *p* = 0.030). Only nine patients (4.4%) required dialysis with a median duration of 14 days (IQR: 13−18 days), while 32 patients (15.6%) required ECMO support with a median duration of 15 days (IQR: 10−19 days).

### 3.4. Weekly Incident Pattern of ICU Patients with COVID-19

**Figure 2:**
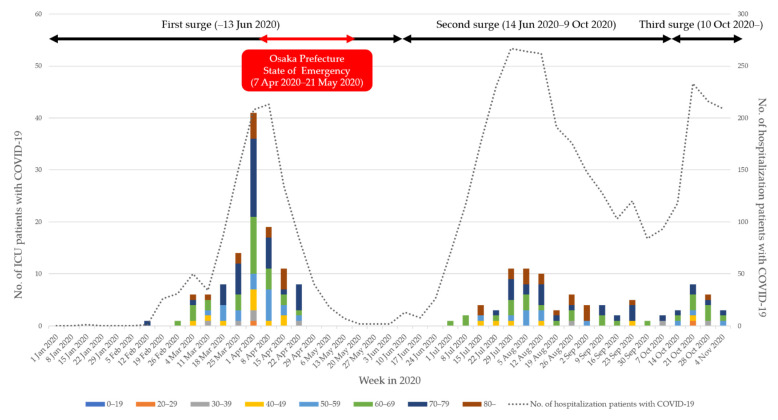
Weekly count of intensive care unit patients with COVID-19 per age group in Osaka, Japan.

Figure 2 shows the weekly count of ICU patients with COVID-19. The highest number of patients with COVID-19 was during week 14 (42 cases; 1–7 April 2020). The total number of ICU patients was 116 during the first surge until 13 June, while 72 patients were admitted to the ICU during the second surge (14 June−19 October 2020).

### 3.5. Factors Related to Mortality

A total of 203 ICU patients were recruited in the Cox regression analysis. Two patients were excluded because the date of ICU admission was unknown or the discrepancy between the date of ICU admission and end of follow-up. Of these 203 patients, 143 were hospital discharged alive, two were released alive after hospital discharge, and 58 were died at the end of follow-up.

The HRs of factors at baseline among patients admitted to the ICU are shown in Table 3. In the univariable analysis, old age was associated with a high mortality rate, and this trend was also observed in the multivariable analysis. The HR for mortality among patients aged >80 years was 6.02 (95% confidence interval: 2.10−17.25), which was higher than that among those aged ≤59 years. Compared with patients without comorbidities, patients with comorbidities had a significantly higher mortality rate (HR: 1.83; 95% CI: 1.04−3.22), but the significance disappeared after adjustment of multivariable factors (HR: 1.43; 95% CI: 0.76−2.69). Gender, demographics, residence, symptoms at diagnosis, surge, cluster, and close contacts were not significantly associated with mortality. The follow-up status of patients at each time point from the onset date is shown in Table S1.

## 4. Discussion

In this study, we analyzed data of 205 patients admitted to the ICU with COVID-19 during the first 10 months of the pandemic in Osaka Prefecture, using a regional database covering the second largest city in Japan. Among these, patients were predominantly men, >70% were over 60 years of age, >60% were from Osaka City, almost 60% had comorbidities, and >90% received some respiratory support (invasive or non-invasive). We believe that clarifying the actual situation of the characteristics and outcomes of ICU patients with COVID-19 using this regional registration of Osaka Prefecture would be helpful and useful for COVID-19 treatment among severe or critical cases in the future.

The overall in-hospital mortality rate was 28.3%, which aligns with those in previous reports of critically ill COVID-19 patients from other countries and regions [3,4,5]. The mortality rate was 59.4% (19/32) among patients aged >80 years, while no deaths were observed among patients aged <39 years. The mortality rate of patients with comorbidities was 35.0%, and that of patients with moderate and severe symptoms at diagnosis was 36.1%. The mortality rate of COVID-19 patients under MV was 28.3%, which was notably lower than that reported in other countries (61.5% in China, 53.4% in Italy, 50% in Seattle, 49.4% in the UK, and 43.6% in New York, USA) [5,15,16,17,18]. In a nationwide study of 217 patients with COVID-19 who were admitted to the ICU and had a defined hospital outcome in Norway, the cumulative overall mortality rate was 18.4%. The mortality rate was 60.0% in patients over 80 years of age, 25.7% in patients with comorbidities, and 20.3% in patients under MV [4]. In a nationwide study in Germany, the COVID-19-related ICU mortality rate was 47% overall, and 57% among patients under MV [3]. Though our results were similar to those of the Norwegian cohort, the mortality rate of patients under MV in the ICU was relatively lower than that reported in most previous studies [4]. Comparisons with other cohorts are challenging because of the scarcity of nationwide data and the lack of international standard criteria for assessing disease severity, the need for ICU admission, and case definitions. Therefore, a global standardized registration on disease severity and ICU admission is needed, which may help lower the mortality rate.

This study highlighted that the mortality rate of COVID-19 patients in the ICU increased with age. In a Norwegian cohort study, the mortality rate increased from 23.8% among patients aged 65−79 years to 53.3% among patients aged ≥80 years [4]. Moreover, patients aged ≥80 years had a shorter survival than those aged 25−49 years [4]. In a German study, patients aged ≥75 years were more likely to have a shorter survival than those aged ≤64 years [3]. Age was associated with mortality among ICU patients with COVID-19 in Osaka Prefecture, Japan; this is in line with findings from previously published studies [3,4,5]. However, we did not have data of the exact age at diagnosis; hence, the mean age could not be calculated and, consequently, could not be compared with data from countries.

Besides age, the presence of comorbidities is another potential factor associated with mortality among ICU patients with COVID-19 in some previous studies. In a Norwegian cohort study, the mortality rate was 22.4% among ICU patients with comorbidities. Further, more ICU patients with comorbidities died than those without [4]. In an Italian study, the mortality was significantly higher among ICU patients with comorbidities (COPD or hypercholesterolemia) than among those without [5]. Our findings confirm that the mortality of critically ill patients with COVID-19 was relatively high among patients with pre-existing comorbidities in the univariable model. Though the significant association disappeared in the multivariable model, a relatively high mortality rate was still observed; nevertheless, comorbidities are considered to be an important factor related to mortality among ICU patients with COVID-19. However, limited by the absence of information on exact diseases, we could not estimate the mortality of each comorbidity in our study; this needs further study.

The number of ICU patients and the proportion of ICU patients among the hospitalized ones during the first surge were higher than those during the second in Osaka Prefecture. This result can be partly explained by the following two possible reasons. First, awareness and behavior among the Japanese population have changed since the first surge of the COVID-19 pandemic. The government appealed to citizens, especially the elderly having higher risks of developing severe or critical symptoms, not to go out unnecessarily [19]. Second, according to various findings from clinical data accumulated owing to the rapidly increasing number of COVID-19 patients during the first surge, treatment strategies, as well as medical systems for COVID-19, might have been well-organized during the second surge. The high number of ICU admissions in the first surge indicates that the healthcare delivery system in Osaka Prefecture had been forced into a difficult situation. Medical facilities across Osaka Prefecture were struggling to cope with the first surge, stretching the medical care system to the brink of collapse. The peak in the first surge occurred during 1 April−7 April. Since then, the Osaka Prefecture State of Emergency was declared on 7 April and lasted until 21 May. It is necessary to monitor the fourth surge, which is expected to start in the spring of 2021.

In a previous study, 10.7% of COVID-19 patients required admission to the ICU during hospitalization up to early July 2020 in Japan, and the median length of ICU stay was 10 days [7]. Of these ICU patients, 64.5% required MV or ECMO [7]. Though the proportion of ICU patients among hospitalized patients in our study was about half of that reported in a Japanese nationwide study, the length of ICU admission was similar [7]. Notably, the proportion of patients requiring respiratory support, including MV and ECMO in our study, was higher than that in a previous Japanese study [7]. In a German study, 81% of patients requiring ICU admissions required invasive MV (median duration, 17 days), and 33% required ECMO support (median duration, 11 days) [3]. In the Norwegian study, the proportion of patients requiring MV was 86.2%, with a median length of 12.0 days [4]. However, only 0.9% of ICU patients required ECMO [4]. In an Italian study, 68.3% of the patients required MV with a median length of 10 days [5]. Thus, ICU admission and ECMO use varied across regions and countries.

Importantly, this study did not include factors related to treatments in Cox regression analysis, as in previous studies because of the possibility of the inversion phenomenon of the cause and effect [15]. Because MV and ECMO were required for severe patients who failed to improve with conventional treatments, it is difficult to observe the relationship between the treatment effect and mortality in observational studies. Though randomized controlled trials have indicated the effectiveness of dexamethasone and remdesivir in COVID-19 patients, further studies are essential to verify the effect of various treatments on Japanese COVID-19 patients who were admitted to the ICU [20,21].

This study has some limitations. First, since this study was an analysis of the epidemiological data collected by public health centers based on the Infectious Diseases Control Law, information on the details of comorbidities and medications was, therefore, unavailable. Second, the data provided by Osaka Prefecture did not include all cases. Therefore, the number of cases, treatments, and other information available on the Osaka Prefecture website were different from our results. Third, as described above, detailed information on age and comorbidities was not collected in the database. Finally, unmeasured confounders might have influenced the results of the multivariable analysis. Despite these limitations, this study accumulating epidemiological information on COVID-19 patients across Osaka Prefecture is important as a fundamental material related to COVID-19 patients requiring ICU admission.

## 5. Conclusions

In this retrospective study, we described the demographic and clinical characteristics of patients admitted to the ICU in Osaka Prefecture, Japan. The mortality rate after ICU admission was 28.3%, and old age was found as the only significant factor related to mortality.

## Figures and Tables

**Table 1:** Epidemiological characteristics of patients admitted to the intensive care unit in Osaka, Japan.

Factor	Total	Survivors		Deaths		*p*-Value
	N	N	(%)	N	(%)	
No. of patients	205	147	(71.7)	58	(28.3)	
Sex						0.584 ^a^
Men	161	114	(70.8)	47	(29.2)	
Women	44	33	(75.0)	11	(25.0)	
Age group, y						<0.001 ^b^
0–19	1	1	(100.0)	0	(0.0)	
20–29	2	2	(100.0)	0	(0.0)	
30–39	8	8	(100.0)	0	(0.0)	
40–49	16	14	(87.5)	2	(12.5)	
50–59	29	26	(89.7)	3	(10.3)	
60–69	53	42	(79.2)	11	(20.8)	
70–79	64	41	(64.1)	23	(35.9)	
≥80	32	13	(40.6)	19	(59.4)	
Geographic area						0.814 ^b^
Osaka City	128	92	(71.9)	36	(28.1)	
Other areas in Osaka Prefecture	73	51	(69.9)	22	(30.1)	
Outside Osaka Prefecture	3	3	(100.0)	0	(0.0)	
Unknown	1	1	(100.0)	0	(0.0)	
Comorbidities						0.013 ^a^
No	88	71	(80.7)	17	(19.3)	
Yes	117	76	(65.0)	41	(35.0)	
Symptoms at diagnosis						0.010 ^b^
Asymptomatic	0	0	NA	0	NA	
Mild	131	101	(77.1)	30	(22.9)	
Moderate	3	1	(33.3)	2	(66.7)	
Severe	69	45	(65.2)	24	(34.8)	
Unknown	2	0	(0.0)	2	(100.0)	
Surge						
First (~13 June)	116	77	(66.4)	39	(33.6)	0.120 ^a^
Second (14 June ~9 October)	67	54	(80.6)	13	(19.4)	
Third (10 October~)	22	16	(72.7)	6	(27.3)	
Cluster						
No	203	146	(71.9)	57	(28.1)	0.487 ^b^
Yes	2	1	(50.0)	1	(50.0)	
Close contact						
No	173	126	(72.8)	47	(27.2)	0.406 ^a^
Yes	32	21	(65.6)	11	(34.4)	

NA, not available. ^a^ Groups compared using Pearson’ s chi-squared test. ^b^ Groups compared using Fisher’ s exact test.

**Table 2:** Clinical characteristics of patients admitted to the intensive care unit in Osaka, Japan.

Factor	Total		Survivors		Deaths		*p*-Value
No. of patients, n (%)	205		147	(71.7)	58	(28.3)	
Days from onset date to ICU admission, median days (IQR)	10	(7, 12)	10	(8, 12)	8	(5, 11)	0.023 ^a^
Length of ICU stay, median days (IQR)	14	(9, 25)	13	(9, 21)	18	(9, 28)	0.177 ^a^
Oxygen therapy, n (%)							
No	9		4	(44.4)	5	(55.6)	0.063 ^b^
Yes	196		143	(73.0)	53	(27.0)	
Length of oxygen therapy, median days (IQR) ^c^	16	(10, 27)	14.5	(10, 27)	18	(10, 26)	0.373 ^a^
Mechanical ventilator ^d^, n (%)							
No	18		13	(72.2)	5	(27.8)	0.960 ^b^
Yes	187		134	(71.7)	53	(28.3)	
Length of mechanical ventilator, median days (IQR)	13	(8, 23)	13	(8, 22)	17	(10, 26.5)	0.030 ^a^
Dialysis, n (%)							
No	196		143	(73.0)	53	(27.0)	0.063 ^b^
Yes	9		4	(44.4)	5	(55.6)	
Length of tracheal intubation, median days (IQR)	14	(13, 18)	14	(13, 14)	18	(12, 21)	0.507 ^a^
Extracorporeal membrane oxygenation, n (%)							
No	173		125	(72.3)	48	(27.7)	0.690 ^b^
Yes	32		22	(68.8)	10	(31.3)	
Length of ECMO, median days (IQR)	15	(10, 19)	15	(10, 17.5)	12	(2, 20)	0.705 ^a^

ICU, intensive care unit; IQR, interquartile range; ECMO, extracorporeal membrane oxygenation. ^a^ Groups compared using Wilcoxon rank-sum test. ^b^ Groups compared using Pearson’ s chi-squared test. ^c^ Number of individuals with missing data of the following variables: length of oxygen therapy *n* = 23; length of mechanical ventilator *n* = 25; length of tracheal intubation *n* = 26; length of dialysis = 3; length of ECMO *n* = 3. ^d^ Mechanical ventilator including invasive and non-invasive positive pressure ventilation.

**Table 3:** Cox regression analysis of factors and mortality among intensive care unit patients in Osaka, Japan.

Variables	No. of Deaths	Person-Days	Crude Rate	Univariable		Multivariable	
			(Per 100)	HR	(95% CI)	HR	(95% CI)
Sex							
Men	47	4449	1.06	Reference		Reference	
Women	11	1429	0.77	0.75	(0.36–1.46)	0.60	(0.30–1.22)
Age group, y							
0–59	5	1320	0.38	Reference		Reference	
60–69	11	1719	0.64	1.74	(0.60–5.03)	2.00	(0.68–5.84)
70–79	23	1829	1.26	3.03	(1.15–8.02)	3.13	(1.15–8.48)
≥80	19	1010	1.88	4.84	(1.80–12.99)	6.02	(2.10–17.25)
Residence							
Osaka City	36	3685	0.98	Reference		Reference	
Other cities	22	2193	1.00	1.04	(0.61–1.77)	1.34	(0.74–2.42)
Comorbidities							
No	17	2447	0.69	Reference		Reference	
Yes	41	3431	1.19	1.83	(1.04–3.22)	1.43	(0.76–2.69)
Symptoms at diagnosis, n (%)							
Mild	30	3445	0.87	Reference		Reference	
Moderate~Severe	26	2377	1.09	1.34	(0.79–2.27)	1.27	(0.72–2.24)
Unknown	2	56	3.57	3.91	(0.93–16.43)	2.49	(0.53–11.64)
Surge							
First (~13 June)	39	3717	1.05	Reference		Reference	
Second (14 June~9 October)	13	1697	0.77	0.74	(0.39–1.39)	0.56	(0.28–1.10)
Third (10 October~)	6	464	1.29	1.19	(0.50–2.83)	1.43	(0.58–3.54)
Cluster							
No	57	5844	0.98	Reference		Reference	
Yes	1	34	2.94	3.26	(0.45–23.92)	4.23	(0.49–36.41)
Close contact							
No	47	5006	0.94	Reference		Reference	
Yes	11	872	1.26	1.31	(0.68–2.54)	1.20	(0.58–2.45)

HR, hazard ratio; CI, confidence interval.

## Data Availability

Data sharing not applicable.

## Supplementary Materials

The following is available online at https://www.mdpi.com/article/10.3390/jcm10112477/s1, Table S1: Follow-up status of the patients at each period from the onset date.

## References

[1] Zhou F., Yu T., Du R., Fan G., Liu Y., Liu Z., Xiang J., Wang Y., Song B., Gu X. (2020). Clinical course and risk factors for mortality of adult inpatients with COVID-19 in Wuhan, China: A retrospective cohort study. Lancet.

[2] Covid-19 Coronavirus Pandemic. https://www.worldometers.info/coronavirus/.

[3] Rieg S., von Cube M., Kalbhenn J., Utzolino S., Pernice K., Bechet L., Baur J., Lang C.N., Wagner D., Wolkewitz M. (2020). COVID-19 in-hospital mortality and mode of death in a dynamic and non-restricted tertiary care model in Germany. PLoS ONE.

[4] Laake J.H., Buanes E.A., Småstuen M.C., Kvåle R., Olsen B.F., Rustøen T., Strand K., Sørensen V., Hofsø K. (2021). Characteristics, management and survival of ICU patients with coronavirus disease-19 in Norway, March-June 2020. A prospective observational study. Acta Anaesthesiol. Scand..

[5] Grasselli G., Greco M., Zanella A., Albano G., Antonelli M., Bellani G., Bonanomi E., Cabrini L., Carlesso E., Castelli G. (2020). Risk factors associated with mortality among patients with COVID-19 in intensive care units in Lombardy, Italy. JAMA Intern. Med..

[6] COVID-19 Bulletin Board. https://covid-2019.live/?from=groupmessage.

[7] Matsunaga N., Hayakawa K., Terada M., Ohtsu H., Asai Y., Tsuzuki S., Suzuki S., Toyoda A., Suzuki K., Endo M. (2020). Clinical Epidemiology of Hospitalized Patients With Coronavirus Disease 2019 (COVID-19) in Japan: Report of the COVID-19 Registry Japan. Clin. Infect. Dis..

[8] Osaka Prefectural Government Press Release on Providing New Coronavirus Patient Data to Academic Research Institutes. http://www.pref.osaka.lg.jp/hodo/index.php?site=fumin&Pageid=39707.

[9] (2021). Ministry of Health, Labour and Welfare Treatment guidelines Version 4.2 of COVID-19 Infection. https://www.mhlw.go.jp/content/000742297.pdf.

[10] Osaka Prefectural Government Monthly Estimated Population of Osaka Prefecture. http://www.pref.osaka.lg.jp/toukei/jinkou/.

[11] Hirayama A., Masui J., Murayama A., Fujita S., Okamoto J., Tanaka J., Hirayama T., Ohara T., Hoffmann E.N., Zhang J. (2021). The characteristics and clinical course of patients with COVID-19 who received invasive mechanical ventilation in Osaka, Japan. Int. J. Infect. Dis..

[12] Osaka Prefectural Government Osaka Prefecture New Coronavirus Countermeasures Headquarters Meeting. http://www.pref.osaka.lg.jp/kikaku_keikaku/sarscov2/.

[13] National Institute of Infectious Diseases Guidelines for Conducting Active Epidemiological Surveillance of Patients with Novel Coronavirus Infections. https://www.niid.go.jp/niid/images/epi/corona/COVID19-02-210108.pdf.

[14] Takeuchi T., Imanaka T., Katayama Y., Kitamura T., Sobue T., Shimazu T. (2020). Profile of patients with novel coronavirus disease 2019 (COVID-19) in Osaka Prefecture, Japan: A population-based descriptive study. J. Clin. Med..

[15] Yang X., Yu Y., Xu J., Shu H., Liu H., Wu Y., Zhang L., Yu Z., Fang M., Yu T. (2020). Clinical course and outcomes of critically ill patients with SARS-CoV-2 pneumonia in Wuhan, China: A single-centered, retrospective, observational study. Lancet Respir. Med..

[16] Bhatraju P.K., Ghassemieh B.J., Nichols M., Kim R., Jerome K.R., Nalla A.K., Greninger A.L., Pipavath S., Wurfel M.M., Evans L. (2020). Covid-19 in critically ill patients in the Seattle region—case series. N. Engl. J. Med..

[17] House N., Holborn H., Wc L. (2020). ICNARC report on COVID-19 in critical care. ICNARC.

[18] Argenziano M.G., Bruce S.L., Slater C.L., Tiao J.R., Baldwin M.R., Barr R.G., Chang B.P., Chau K.H., Choi J.J., Gavin N. (2020). Characterization and clinical course of 1000 patients with coronavirus disease 2019 in New York: Retrospective case series. BMJ.

[19] Takeuchi T., Kitamura T., Hirayama A., Katayama Y., Shimazu T., Sobue T. (2020). Characteristics of patients with novel coronavirus disease (COVID-19) during the first surge versus the second surge of infections in Osaka Prefecture, Japan. Glob. Health Med..

[20] RECOVERY Collaborative Group (2020). Dexamethasone in hospitalized patients with Covid-19—preliminary report. N. Engl. J. Med..

[21] Beigel J.H., Tomashek K.M., Dodd L.E., Mehta A.K., Zingman B.S., Kalil A.C., Hohmann E., Chu H.Y., Luetkemeyer A., Kline S. (2020). Remdesivir for the treatment of Covid-19. N. Engl. J. Med..

