# Supplementary material for: Characteristics and Survival of Intensive Care Unit Patients with Coronavirus Disease in Osaka, Japan: A Retrospective Observational Study
Source: J Clin Med. 2021 Jun 3;10(11):2477. doi: 10.3390/jcm10112477 (PMC8199743; doi:10.3390/jcm10112477)
Supplement: Supplementary file 1 [file jcm-10-02477-s001.zip › jcm-1212044-supplementary.pdf]

Table S1. Follow-up status of the patients at each period from the onset date.

Period from the onset date	10 days		20 days		30 days		40 days		50 days		60 days		70 days		80 days		90 days		106 days		138 days	
	**N**	**(%)**	**N**	**(%)**	**N**	**(%)**	**N**	**(%)**	**N**	**(%)**	**N**	**(%)**	**N**	**(%)**	**N**	**(%)**	**N**	**(%)**	**N**	**(%)**	**N**	**(%)**
Survivors under follow-up	185	(91.1)	128	(63.1)	73	(36.0)	37	(18.2)	23	(11.3)	12	(5.9)	11	(5.4)	9	(4.4)	8	(3.9)	3	(1.5)	0	(0.0)
Subjects with hospital discharged alive or released alive	6	(3.0)	48	(23.6)	85	(41.9)	114	(56.2)	124	(61.1)	134	(66.0)	135	(66.5)	137	(67.5)	138	(68.0)	142	(70.0)	145	(71.4)
Deaths	12	(5.9)	27	(13.3)	45	(22.2)	52	(25.6)	56	(27.6)	57	(28.1)	57	(28.1)	57	(28.1)	57	(28.1)	58	(28.6)	58	(28.6)
Total[a]	203	(100.0)	203	(100.0)	203	(100.0)	203	(100.0)	203	(100.0)	203	(100.0)	203	(100.0)	203	(100.0)	203	(100.0)	203	(100.0)	203	(100.0)

[a] Of the 205 patients who were admitted to the intensive care unit, two patients were not recruited in the Cox regression analysis because the date of ICU admission was unknown or the discrepancy between the date of ICU admission and end of follow-up.
